# Knowledge, attitudes, and practices (KAP) during the malaria elimination phase: A household-based cross-sectional survey

**DOI:** 10.1097/MD.0000000000033793

**Published:** 2023-06-02

**Authors:** Siddig Ibrahim Abdelwahab, Ibrahim M. Elhassan, Osama Albasheer, Manal Mohamed Elhassan Taha, Nasir Ahmed Ali, Yahya Salem Al-Jabiri, Waleed Madkhali, Ahmad A. Sahly, Bassem Oraibi, Ahmed Abdallah Ahmed Altraifi, Nasser Hakami, Mohammed M. Alshehri, Mohammad Abu Shaphe, Rashid Ali Beg, Meshal Alshamrani

**Affiliations:** a Medical Research Centre, Jazan University, Jazan, Saudi Arabia; b Institute of Endemic Diseases, University of Khartoum, Khartoum, Sudan; c Family and Community Medicine Department, College of Medicine, Jazan University, Jazan, Saudi Arabia; d Medical Research Centre, Jazan University, Jazan, Saudi Arabia; e College of Public Health and Tropical Medicine, Jazan University, Jazan, Saudi Arabia; f Medical Research Centre, Jazan University, Jazan, Saudi Arabia; g Medical Research Centre, Jazan University, Jazan, Saudi Arabia; h Department of Vector Borne Diseases, Ministry of Health, Jazan, Saudi Arabia; i Medical Research Centre, Jazan University, Jazan, Saudi Arabia; j Obstetrics and Gynecological Department, College of Medicine, Jazan University, Jazan, Saudi Arabia; k Surgical Department, College of Medicine, Jazan University, Jazan, Saudi Arabia; l Medical Research Center, Jazan University, Jazan, Saudi Arabia; m Department of Physical Therapy, College of Applied Medical Sciences, Jazan University, Jazan, Saudi Arabia; n Department of Pharmaceutics, College of Pharmacy, Jazan University, Jazan, Saudi Arabia.

**Keywords:** attitudes, elimination program, knowledge, malaria, practices, Saudi Arabia

## Abstract

Malaria is a major health problem in Southwestern Saudi Arabia. This study aimed to measure the level of community understanding of malaria transmission, protection, and treatment. A questionnaire-based cross-sectional study enrolled 1070 participants from 2 districts with different malaria prevalence rates in Jazan Province. The response rate was 97.27%. Of the 1070 total; 754 (70.5%) had heard about malaria. Sixty-seven percentage know that fever was the main symptom. Approximately 59.8% did not know that stagnant water is one of the most important locations for mosquito breeding. Nevertheless, we found that 50% of the participants knew that mosquitoes bite at night and 96.9% confirmed that mosquitoes did not bite during the day. The most effective sources of information were distributed leaflets (41.8%) and video awareness (31.9%). The most significant factors affecting participants knowledge were gender, residence, family members, income, and education (*P* < .05). Knowledge levels were satisfactory in this study, and the majority of participants exhibited adequate attitudes and practices related to malaria prevention. However, knowledge differences were observed with regard to the place of residence. Greater emphasis should be directed towards education programs in malaria-endemic areas to ensure complete eradication of malaria.

## 1. Introduction

Despite decades of malaria prevention initiatives, malaria remains a severe public health concern worldwide.^[1]^ It is a leading cause of mortality and morbidity in many developing countries, where young children and pregnant women are most affected.^[2]^ In 2016, malaria triggered 216 million clinical episodes and 445,000 deaths worldwide.^[3]^ The Global Technical Strategy for Malaria 2016 to 2030 is designed to guide and support all malaria-affected countries. The strategy targeted for the year 2030 an elimination of malaria in at least 35 countries and reduction of malaria case incidence and mortality rate by at least 90%.^[2]^ The revived enthusiasm for scaling up the adoption of validated strategies around the world, however, has rekindled optimism, and even elimination is being pursued where malaria has been reduced to very low levels.^[4,5]^

The only 2 countries on the Arabian Peninsula that have not yet eradicated malaria are Yemen and Kingdom Saudi Arabia.^[6,7]^ Saudi Arabia joined the World Health Organization global malaria eradication effort and the recommendations on malaria prevention and control were updated based on the latest available evidence. Its achievements include biannual meetings and the use of mobile units to assist undocumented refugees arriving from neighboring countries of the Autonomous Country Committee on Malaria Elimination. Saudi Arabia needs to improve vector surveillance along its frontier and expand the number of specialists employed in the entomology and case management of malaria to achieve zero.^[6–10]^

Malaria elimination is defined as the purposeful interruption of local transmission of a particular malarial parasite species in a defined geographical region as a consequence of deliberate operations. This resulted in a reduction in the incidence of indigenous malaria. It is necessary to maintain preventative measures that have been taken to stop the reestablishment of transmission. For a nation to receive official recognition that the disease has been eradicated, the local transmission of all human malaria parasites must first be stopped. It is necessary to take precautions against the resumption of transmission indefinitely, until the disease has been eradicated.^[2,11–14]^ It is essential to have excellent surveillance and response systems in place in order to eliminate and keep the malaria problem under control; information systems need to become more “granular” in order to enable the identification, tracking, classification, and response to each and every case of malaria (imported, introduced, and indigenous).^[12–16]^ For a country to be able to eliminate the disease, its healthcare system must have both capable leadership and the ability to penetrate all of the country’s communities (e.g., with systems to ensure access, track progress, deliver quality services, and rapidly and effectively respond to epidemiological challenges). Knowledge should be generated through operational research on tools, methods, and delivery and used to enhance recommendations and future elimination initiatives.^[12–14,16,17]^

Over the last 50 years, great efforts to control malaria in Saudi Arabia have led to a decrease in its prevalence in many parts of the country, mainly by using insecticide spraying, treatment, and personal protection measures.^[7,18,19]^ Malaria remained at a low level in the Kingdom’s southern regions.^[18]^ These governmental efforts to fight malaria require strong collaboration and pioneering public awareness. Parallel research on knowledge, attitude and practice (KAP) must be conducted to measure public bonding. A previous study of KAP in the Jazan region did not focus on endemic areas and compared them with disease-free areas, as was the case in the present study. In addition, this study includes an objective evaluation of what happened after elimination, and the results will be useful to relevant authorities and decision-makers.^[4]^Therefore, the current study was designed to measure the level of community understanding of malaria transmission, personal protection, and treatment, to determine the level of KAP in the community for the concepts of control and elimination, and to compare the knowledge of families in 2 regions characterized by different prevalence rates during the period of the National Malaria Elimination Program.

## 2. Materials and Methods

### 2.1. Study area and population

**Figure 1.**
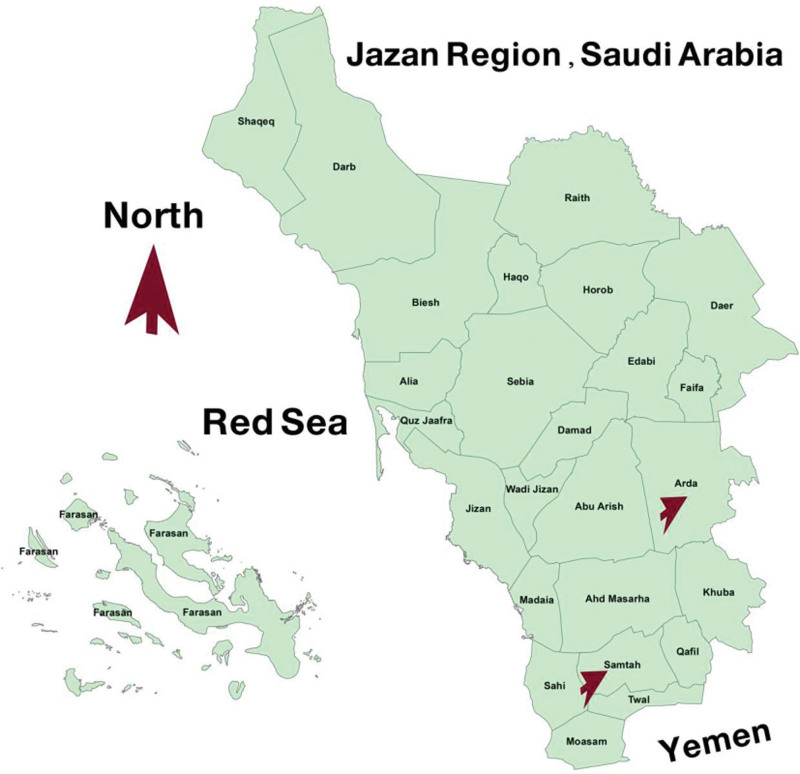
Map of Jazan Region.

This research was conducted in 2 districts with different malaria prevalence rates in the Jazan Province (Al-Ardah and Samtah Regions).^[20]^ The province of Jazan stretches 300 km along the southern Red Sea coast and 120 km along the border with Yemen. According to the 2010 census, there were 1.37 million inhabitants. A map of Jazan is shown in Figure 1. Multistage random sampling was used to divide the population and select households. The Al-Ardah Region is composed of 3 main centers with a total of 84 villages and around 10 thousand households. The Samtah Region is composed of 2 main centers, with a total of 39 villages and around 30 thousand households. A total of 13 villages (9 villages from Al-Ardah and 4 villages from Samtah) were selected randomly from these centers (subgroups). Each village was listed with a number, and a starting point was selected randomly from the first 10 listed villages, from which every 10^th^ village on the list was selected. The same randomization was applied for the selection of households, with 4 hundred households selected in total. We interviewed 2 to 3 people from each household and the total number of people interviewed was 1100.

### 2.2. Study design, data collection, and period

This is a cross-sectional study. Data were collected using a well-structured questionnaire distributed between February and April 2021. A comprehensive workshop on how to use the questionnaire was conducted by the authors. The data collectors were final-year students from the Department of Health Education and Promotion, Faculty of Public Health and Tropical Medicine, Jazan University, Kingdom of Saudi Arabia.

### 2.3. Sample size and sampling

Based on the sample size guidelines for logistic regression from observational studies with large populations, the minimum sample size to determine the associated factors was 500, assuming differences within ± 0.5 for coefficients and ± 0.02 for Nagelkerke r-squared.^[21]^ For the large population in this study, a total sample of 1100 people was collected assuming differences within ± 1.0 for coefficients and ± 0.02 for Nagelkerke r-squared to derive the statistics that represent the parameters.

### 2.4. Study measures and questionnaire design

The structured questionnaire included demographic characteristics in the first part (nationality, gender, social status, number of family members, income, education level, type of accommodation, and area). The second part contained questions about knowledge, attitudes, and practices, which were designed based on previous studies^[4,22–30]^ and World Health Organization guidelines.^[2,11,15]^ The knowledge questions were about the causes of malaria, the primary vector, symptoms, preventive measures, personal means for prevention, and knowledge about mosquito behavior. The main question for measuring knowledge of malaria consisted of 4 options: no information, little information, medium information, and good information. This assumption was based on assessments of the data collectors. Heard about malaria and know about its transmission is the minimum requirement to categorize respondents into little information. If the respondent knew about the transmission and symptoms, they were placed in the moderate information category. If the respondent’s knowledge extended to the treatment of malaria, it was placed in the good information category. Based on these choices, the knowledge prevalence rate was calculated, and this variable was used as the dependent variable in multivariate logistic regression analysis. Data on mosquito breeding areas were also collected. The questionnaire also had a section that asked about people’s habits, such as where they slept, how they cleaned their water containers, covered their tanks, used insecticides, and shut their windows.

### 2.5. Pilot study

The questionnaire was translated into Arabic and pretested in a pilot study (n = 30) before the final analysis. The respondents for the pilot study were selected from 2 villages (1 village from each region), and 15 respondents were chosen from 15 households in each village. Trained interviewers conducted interviews with responsible adults in their respective families. Only 1 individual per household was interviewed in the pilot study. Verbal permission was granted to all participants. For quality management, questionnaire administration and data collection were tracked regularly. Data obtained from the pilot study were not included in the final analysis.

### 2.6. Data management and analysis

Data were entered into an Excel file and transferred to the SPSS statistical software version 20.0 (IBM, USA), where all descriptive and inferential statistics were obtained. The reliability of the questionnaire was tested using a Cronbach alpha of 0.713. Binary logistic regression modeling was performed with knowledge as the dependent variable (0 = no information; 1 = information) and demographic factors as independent variables. As few respondents were in the moderate and good categories for knowledge, we placed them with the little category in 1 group and compared them with those with no information category in the binary logistic regression modeling. Dummy variables and reference groups for each independent variable were generated using SPSS software. Model fitting was evaluated using the-2 log-likelihood, Cox & Snell R-Square, Nagelkerke R-Square, and Hosmer and Lemeshow tests.^[31]^

### 2.7. Institutional review board statement

“The study was conducted in accordance with the Declaration of Helsinki, and approved by the Institutional Review Board (or Ethics Committee) of the Department of Health Education and Promotion, Faculty of Public Health and Tropical Medicine, Jazan University, Saudi Arabia. (Protocol code FSP-2/194 on 10/12/2020)”.

## 3. Results

### 3.1. Baseline characteristics

The data collection process had a 97.27% response rate. Table 1 presents the demographic characteristics. The current study included 49.8% (n = 533) men and 50.2% (n = 537) women. The respondents mean age is 24.26 ± 5.0 years, with a median of 19.0 years. The minimum and maximum age was 18 and 66 years, respectively. The majority of participants (n = 1053, 98.4%) were Saudis, and 17 (1.6%) were non-Saudi. The study sample comprised of 66.3% (n = 709) single people and 31.8% (n = 340) married people. The number of family members varied in this study, with families with fewer than 5 members accounting for 57.1% (n = 611) of the study population. Families with 5 to 10 members account for 42.8% of the sample (n = 458). In terms of monthly financial income, 50.3% of participants had a monthly income ranging from 5000 to 10,000 Saudi riyals. The percentages for categories 5000 to 10,000 and 1000 to 15,000 were approximately equal. The sample comprised 49.2% buildings and 29.5% traditional houses. The additional details are presented in Table 1.

**Table 1:** Demographic characteristics.

Variables	Statistics	
	Frequency	%
Nationality		
Saudi	1053	98.4
Non-Saudi	17	1.6
Gender		
Male	533	49.8
Female	537	50.2
Social status		
Single	709	66.3
Married	340	31.8
Divorced	13	1.2
Widowed	8	.7
Number of family members		
<5	611	57.1
5–10	458	42.8
More than 10	1	.1
Income		
<5000	278	26.0
5000–1000	538	50.3
1000–15,000	253	23.6
More than 15,000	1	.1
Education level		
Uneducated	38	3.6
Average or less	57	5.3
Secondary school	657	61.4
Diploma	67	6.3
Bachelor	245	22.9
Postgraduate	6	.6
Accommodation type		
Popular house	316	29.5
Flat	164	15.3
Building	526	49.2
Villa	64	6.0
Area		
Al-Ahrdah	611	57.2
Samtah	458	42.8
Total	1070	100

### 3.2. Malaria knowledge-transmission, causes, and symptoms

**Figure 2.**
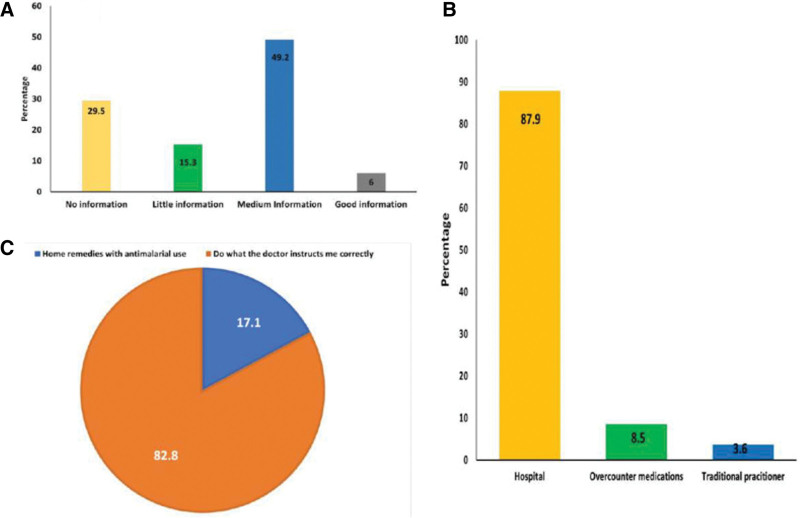
(A) Respondents’ knowledge of malaria. Y-axis represents percentage of the participants. (B) Behavior of participant when a family member infested with malaria. (C) Respondents’ opinion on the correct method of malaria’s treatment.

Of the 1070 household members surveyed, 754 (70.5%) had heard of malaria (Fig. 2). Figure 2A presented the 4 categories of malaria knowledge. The respondents scored 29.5%, 15.3%, 49.2%, and 6% for the no, little, moderate, and high information categories, respectively. Knowledge about malaria treatment was high, with 87.9% (n = 940) of the respondents stating that they would seek treatment in a public hospital and 8.5% mentioning over the-counter (OTC) medications. Only 3.6% (n = 39) of the participants sought treatment from traditional healers (Fig. 2B). Figure 2C depicts respondents opinions regarding the correct method of malaria treatment. Eighty-two percentage (886) of the respondents prefer to do what the doctor instructs them correctly, while 17.2% (n = 184) use home remedies with antimalarial use.

Table 2 demonstrates the respondents’ knowledge of the transmission and symptoms of malaria; 23.0% of the respondents answered correctly that the mosquitoes transmit malaria. Other vectors like flies, fleas, ticks and mites were selected as vectors for malaria transmission by 77% of the respondents. Regarding mode of transmission: 508 (47.5%) of respondents were aware of the correct malaria transmission method. Fever was the primary symptom of malaria in 67% of the respondents. Other symptoms such as lack of appetite and sweating, we selected in 5.4 and 5.7% of respondents respectively. Measures for prevention of mosquito bites such as using mosquito nets, shutting windows with an iron net and using insecticides were selected by 19.3%, 26.5%, and 36.8% of the respondents respectively. Regarding prevention of mosquito breeding: measures such as cleaning the perimeter of the house, pulling or unloading stagnant water and cleaning the small shrubs around the house were selected by 45.9%, 27.8% and 24.8% of the respondents respectively.

**Table 2:** Respondents’ knowledge on concept of transmission and symptoms, prevention of malaria.

Variable	N (%)
Vector	
Mosquitoes	246 (23.0 %)
Others (flies, fleas, ticks)	824 (77.0 %)
The concept of transmission	
Mosquitoes bites	287 (26.8 %)
Mosquitoes bites carrying the blood of a person with malaria	508 (47.5 %)
Other factors (stagnant water - unclean environment - climate)	189 (17.7 %)
I do not know	88 (22.6 %)
Symptoms	
Fever	719 (67%)
Sweating	58 (5.4%)
Lack of focus	37 (3.5%)
Anorexia	61 (5.7%)
I do not know	252 (24%)
Personal measures	
Using mosquito nets	313 (29.3%)
Shutting windows with iron net	284 (26.5%)
Using an insecticide	394 (36.8%)
Using pills for prevention	64 (6.0%)
I do not know	222 (20.7%)
Methods of prevention of mosquito breeding	
Cleaning the perimeter of the house	491 (45.9%)
Pull or unload stagnant water	297 (27.8%)
Clean the small shrubs around the house	265 (24.8)
Others	161 (15.0)
Mosquitoes breeding sites	
Stagnant water	430 (40.1%)
Long grass	136 (12.7%)
Small shrubs	183 (17.1%)
Sewage	290 (27.1%)
I don’t know	206 (19.3%)
When mosquito bite is made	
During the night	575 (53.7%)
During the day	33 (3.1%)
Any time	344 (32.1%)
I do not know	118 (11.0%)
Total	1070 (100)

As observed, 59.8% (n = 640) of them did not know that stagnant water was one of the most critical places for mosquito breeding. Nevertheless, 50% knew that mosquitoes bite at night, and 96.9% (n = 1037) confirmed that mosquitoes do not bite during the day, as shown in Table 2.

### 3.3. Perception about effective educational materials and awareness campaigns

Statistics regarding preferred educational resources and the most effective campaigns are listed in Table 3. The distribution of leaflets and video awareness were the most important sources of information, with 41.8% and 31.9%, respectively. Similar results were observed for stickers (8.1%), radio broadcasting (8.9%) and others (9.3%). From the total sample (n = 1070), 47.1% believed that media campaigns about malaria were periodically followed by home visits, constituting 16.4%. Awareness boards, meetings, and others accounted for 16.1%, 7.4%, and 9.1%, respectively.

**Table 3:** Perception about effective educational materials and awareness campaigns.

Educational materials	N	(%)
Distribution of leaflets	447	(41.8)
Stickers	87	(8.1)
Video awareness	341	31.9
Listen to the radio	95	8.9
Other	100	9.3
Awareness campaigns		
Media campaigns on malaria periodically	504	47.1
Awareness boards	172	16.1
Home visits	218	20.4
Meetings	79	7.4
Others	97	9.1

### 3.4. Practices of the population towards malaria in Jazan rural areas

Table 4 shows the distribution of the participants according to their habits and practices. Among the respondents, 88.5% (n = 950) reported that they did not sleep in the house yard. Moreover, 65% (n = 696) practiced the regular cleaning of water containers. Regarding exposure to water and household usage of pesticides, 87.3% (n = 934) and 61.3% (n = 656) practiced such measures of malaria prevention, respectively. Practices such as closing home windows, allowing rainwater to gather around the house, having small bushes around their homes, and regularly checking blood for malaria were 82.8% (n = 886), 33.0% (n = 356), 68.5% (n = 733), and 12.3% (n = 134), respectively. An inferential analysis was conducted using the chi-square of habits based on the geographical location of the respondents. Some practices showed statistically significant differences between the 2 regions, except for cleaning water containers, closing windows, covering exposed water tanks, closing house windows, leaving rainwater around the house, and blood testing for malaria (Table 4).

**Table 4:** Distribution of participants according to their habits and practices.

Variable	N (%)				Chi-square	*P* value
	Yes		No			
	Al-ardah	Samtah	Al-ardah	Samtah		
Sleeping in the house’s yard	120 (11.2%)		950 (88.8%)		9.47	.002
	53 (5)	67 (6.3)	558 (52.3)	389 (36.5)		
Regularly cleaning water bowls	696 (65.0%)		374 (34.9%)		0.167	.692
	394 (36.9)	302 (28.3)	216 (20.2)	157 (14.7)		
Covering exposed water tanks	934 (87.3%)		136 (12.7%)		0.648	.421
	529 (49.9)	405 (37.9)	82 (7.7)	54 (5.0)		
Regular use of pesticides at home	656 (61.3%)		414 (38.6%)		18.25	.000
	408 (38.2)	248 (23.2)	202 (18.9)	211 (19.7)		
Closing the windows of the house	886 (82.8%)		184 (17.2%)		1.74	.187
	514 (48.0)	372 (34.8)	97 (9.1)	87 (8.1)		
Rainwater around the house for a long time	356 (33.0%)		714 (66.7%)		1.915	.384
	206 (19.3)	147 (13.8)	402 (37.6)	312 (29.2)		
Existence of small bushes around the house	733 (68.5%)		337 (31.5%)		23.4	.00
	455 (42.5)	278 (26.0)	156 (14.6)	181 (16.9)		
Checking blood for malaria on a regular basis	134 (12.3%)		936 (87.5%)		0.979	.322
	70 (6.6)	62 (5.8)	539 (50.5)	397 (37.2)		

### 3.5. Knowledge and perceptions about operational vector control activities

Knowledge of the fight against malaria campaigns was reported by 52.5% (n = 562) of respondents, as shown in Table 5. A total of 69% of the 1070 people polled confirmed that spraying occurred during the malaria transmission season. The headquarters for controlling communicable diseases (malaria) near their homes is known to be 23.9% of the 1070 households sprayed. The participation of rural Jazan residents in anti-malaria programmes could be more encouraging, with only 11.8% participating in such programmes. Knowledge of the means and methods of malaria control among the public is one of the most critical factors, as the current study showed that only 42.9% of respondents knew about them. Similarly, the percentage of participants in these campaigns was low, reaching only 15% (Table 5). An inferential analysis was performed using the chi-square test on the attitudes and behaviors of the respondents depending on their geographical location, and an association with high statistical significance was observed (Table 5).

**Table 5:** Attitudes and practices against malaria elimination program.

Variables	N (%)						Chi-square	*P* value
	Yes		No		I don’t know			
	Al-ardah	Samtah	Al-ardah	Samtah	Al-ardah	Samtah		
Did you hear about the fight against malaria?	562 (52.5)		397 (37.1)		111 (10.4)		39.98	.000
	303 (28.3)	259 (24.2)	268 (25.0)	129 (12.1)	40 (3.7)	71 (6.6)		
Is there an insecticide spraying process in your home?	652 (60.9)		340 (31.8)		78 (7.3)		19.629	.000
	381 (35.6)	271 (25.3)	204 (19.1)	136 (12.7)	26 (2.4)	52 (4.9)		
Do you know the headquarters of the control of communicable diseases (malaria) near your home?	256 (23.9)		673 (62.9)		141 (13.2)		43.131	.000
	149 (39.0)	107 (10.0)	417 (39.0)	256 (23.9)	45 (4.2)	96 (9.0)		
Did you participate in the malaria control process?	126 (11.8)		883 (82.5)		61 (5.7)		6.482	.039
	68 (6.4)	58 (5.4)	517 (48.3)	366 (34.2)	26 (2.4)	35 (3.3)		
Do you know the means and methods of fighting malaria?	459 (42.9)		513 (47.9)		98 (9.2)		41.600	.000
	221 (20.7)	238 (22.2)	345 (32.3)	168 (15.7)	45 (4.2)	53 (5.0)		
Have you been guided or attended any malaria education program?	189 (17.7)		817 (76.4)		64 (6.0)		48.644	.00
	69 (6.4)	120 (11.2)	514 (48.0)	303 (28.3)	28 (2.6)	36 (3.4)		

### 3.6. Logistic regression

Further analysis using binary logistic regression with recategorized knowledge was used as the dependent variable. Respondents with no information (n = 316) were categorized and coded as 0. While 754 (70.5%) respondents who had heard about malaria were classified and coded as 1, the independent variables included in the logistic regression model were gender, age, residence, family members, region, marital status, income, type of home, and education. Beta coefficients, p-values, adjusted, and crude odds ratios (OR) are reported in Table 6. Adjusted and crude odd ratios were obtained using univariate and multivariate logistic regression, respectively. All independent variables in the univariate analysis were significant in their effect on respondents knowledge of malaria, except for residence and home type. The inclusion of these variables in the multivariate model led to a significant change in crude OR, as shown in Table 6. The OR dropped from 0.78 to 0.53 and became statistically significant, indicating that the location had an effective influence on knowledge. People living in villas fall under this category. Age and marital status were 2 characteristics that were shown to have lost their predictive power after being added to the multivariate model.

**Table 6:** Binary logistic regression modeling of knowledge as a dependent variable (0 = no information; 1 = information) with demographic factors as predictors.

Predictors		COR	AOR	CI (95%)	
				Lower	Upper
Home type	Traditional home (Ref)				
	Apartment	1.93	0.85	0.45	1.60
	Building	1.42	1.45	0.41	5.08
	Villa	0.29	0.374*	0.14	.991
	Others	0.51	0.59	0.31	1.13
Education	Student (Ref)				
	Officer	0.90	1.17	.431	3.18
	Ritered	2.06*	2.78	1.059	7.31
	Free lancer	3.79*	2.98	.949	9.35
	No work/job	5.60*	4.28*	1.583	11.57
Income	<5000SAR (Ref)				
	5000–10,000 SAR	2.32*	1.83*	1.296	2.57
	10,000–15,000 SAR	4.37*	4.19*	2.514	6.97
	More than 15,000 SAR	4.15*	3.94*	1.773	8.74
Marital status	Single (Ref)				
	Married	1.47*	0.94	.564	1.56
	Divorced	1.58	0.67	.166	2.72
	Widowed	1.42	2.95	.448	19.40
Family members	<5 members (Ref)				
	5–10 member	0.84	0.76	0.52	1.11
	More than 10	0.59*	0.57*	0.37	0.88
Residence	Al-Ardah (Ref)				
	Samtah	0.78	0.53*	0.40	0.73
Gender	Male (Ref)				
	Female	2.02*	1.78*	1.298	2.44
Age		1.012*	1.02	0.99	1.05
Model fit parameters	−2 Log-likelihood*	Cox & Snell R-Square*	Nagelkerke R-Square*	Chi-square (*P* value) - Hosmer and Lemeshow Test*	
	1154.85	0.125	0.178	7.97 (0.44)	

AOR = adjusted odds ratio, CI = confidence intervals, COR = crude odds ratio.

* statistically significant association.

## 4. Discussion

The goal of the current study was to compare the knowledge of households in 2 regions with different malaria prevalence rates to assess how well the community understood malaria transmission, personal protection, and treatment. It also assessed the extent to which the community understood the concepts of control and elimination. This is the first study in Saudi Arabia to include baseline evidence and an evaluation of knowledge, attitudes, and practices during the malaria elimination phase in Jazan, rural Saudi Arabia.

Despite the existence of effective preventive strategies, the burden of malaria is increasing in many countries.^[15,16]^ Understanding community perspectives and practices is an essential component of a successful malaria control program.^[17]^ In most affected nations, the possible contribution of KAP studies to malaria research and control has received little attention.^[18]^ The findings of this study are directly reflected in public awareness. Of the 1070 household members surveyed, 754 (70.5%) had heard of malaria. These results are in agreement with those of previous studies conducted in Tanzania,^[32]^ western Sierra Leone,^[33]^ and Saudi Arabia.^[4]^ This high level of knowledge about malaria may be due to the work that the Saudi authorities in charge of public health have done on a national level. Learning about malaria is insufficient; rather, it should be viewed as a foundation for understanding a wide variety of malaria-related topics such as transmission, symptoms, prevention, and therapy. More than 2 to 3rd of the respondents in this study were correctly answered the questions related to the malaria symptoms and the method of transmission. However, there was misconception with regard to the vector that transmitted malaria as only 1 to 3rd of the respondents selected mosquitoes as a pathogen vector for malaria. The findings of this study lend credence to those found in earlier research carried out in Nigeria,^[23,34,35]^ Guinea,^[28]^ Tanzania,^[29]^ Ethiopia,^[36]^ and India.^[24]^

This study revealed that most people in both regions had sufficient knowledge of the malaria symptoms. The most frequent symptom, fever, was recognized by more than 2 to 3rd of participants (67%). This finding is consistent with previous research in endemic countries with tropical and subtropical climates.^[9,10]^ It should be noted that there was confusion between malaria symptoms and dengue fever, which is also prevalent in these regions, among many participants in the Al-Ardah region. However, mosquito prevention does not affect disease control. However, this can lead to severe problems in patients who assume that dengue fever may be treated with antipyretics and drinking water.^[25,27]^

Public health is critical for the elimination of malaria.^[7]^ The present study showed variable findings on these measures, such as sleeping behavior, water container cleaning, opening water tanks, use of pesticides, closing windows, rainwater monitoring, cultivation of small shrubs, and regular checking for malaria. This may be because most people rely on malaria prevention programs to defend themselves from malaria infections. These inconsistent statistics should be considered by health authorities, and further actions should be taken based on the deficits depicted in these measures. In the current study, a significant proportion of participants incorrectly identified the primary control measures. Moreover, 72.7% and 63.2% of the participants did not recognize bed nets and insecticides as essential measures for malaria prevention and control, respectively. These are considered to be high figures. Only 20.7% of Americans currently use personal malaria-protection measures. This might be explained by the outstanding efforts of the Ministry of Health, which reduced malaria to minimum levels in previous years. Responses regarding knowledge of malaria symptoms and signs showed that only 8% had good knowledge, and 47% needed more information about the disease. A 2005 study found that participants in India had a moderate-to-high degree of knowledge and successfully implemented preventative actions.^[30]^ Only 4% of participants in a cross-sectional study of 1330 households in rural Nepal conducted in 2004 to 2005 demonstrated any knowledge of the significance of using insecticide-impregnated mosquito nets, and only 23% of these people actually used nets.^[26]^ This Nepali study revealed that participants had a minimal understanding of preventive measures.

Our results are encouraging for malaria treatment. Eighty-two point eight Of the respondents, 82.8% preferred to do what doctors had instructed them correctly. Only 3.6% of participants sought treatment from traditional healers. Previous surveys in Southeast Asian rural communities have shown that over half of the residents prefer to manage their health without attending health centers. The improved conduct documented in this study may be attributed to the provision of health facilities to all Saudi citizens and access to their services. This behavior helps eliminate malaria, as mosquitoes cannot transfer infected blood to healthy people and break the disease transmission cycle.^[37,38]^

Exciting results were obtained from logistic regression modeling of knowledge levels across both regions. Sex, age, home, family members, region, income, and education were independent variables used in the logistic regression model. All of these variables had an important impact on malaria awareness, except for age and marital status. The inclusion of all the variables in the model substantially changed the crude OR. This modeling analysis provided an additional advantage over previous studies^[22,39]^ that used descriptive patterns only for malaria KAP.

A significant difference in the knowledge level of malaria was observed between the villagers from Al-Ardah and Samtah. This could be explained by the differences in climate between the 2 regions. The weather in the Al-Ardah region is tropical and the rainy season is summer. Al-Ardah rains significantly during autumn. This climatic difference between the 2 regions leads to differences in mosquito breeding rates. On the other hand, Al-Ardah is a valley and mountainous area, whereas Samtah is a plain area. Therefore, the environment in the Al-Ardah region is considered suitable for mosquito breeding, as swamps, rain, and water pools are the most important factors that help the spread of mosquitoes. The higher number of health facilities recorded in Samtah may explain this finding.^[40]^

This study presents the first evidence of KAP regarding malaria, and it was conducted in large rural populations that were randomly chosen from well-populated villages in the southwestern region of Saudi Arabia. This region is known to have a comparatively high risk of exposure to imported malaria. However, possible limitations of the research include the use of a biased tool to assess the KAP of participants, as well as the cross-sectional design of the study, which makes it difficult to establish any causative relationship. Both aspects of the study could be considered drawbacks. Despite this, it was a strategic strategy to evaluate the baseline information on KAP in relation to the treatment and prevention of malaria.

## 5. Conclusions

The vast majority of respondents in this study heard about malaria and they know much about its symptoms and mode of transmission. However, there was misconception with regard to the causative agent and there were variations related to the knowledge between the different regions. As a result, we suggest that a collection of local strategies should be developed to encourage the implementation of preventative and control measures that aim to minimize the exposure to, occurrence of, and spread of malaria as well as to encourage positive attitudes and the most effective preventative practices that are currently available. Additional research is necessary to validate and broaden the scope of the findings of this study.

## Acknowledgments

The authors are grateful to the participants of this study. The authors also extend their appreciation to the Deanship of Scientific Research, Jazan University, for supporting this research through the Research Unit Support Program (Support Number: ISP22-7).

## Author contributions

**Conceptualization:** Ibrahim Siddig Abdelwahab, Ibrahim Elhassan, Osama Albasheer.

**Data curation:** Ibrahim Siddig Abdelwahab, Ibrahim Elhassan, Osama Albasheer, Nasir Ali.

**Formal analysis:** Ibrahim Siddig Abdelwahab.

**Investigation:** Waleed Madkhali.

**Methodology:** Ibrahim Siddig Abdelwahab, Ibrahim Elhassan, Mohammed Alshehri.

**Project administration:** Ibrahim Siddig Abdelwahab, Osama Albasheer.

**Resources:** Nasir Ali, Yahya Al-Jabiri, Bassem Oraibi, Ahmed Altraif, Nasser Hakami, Mohammed Alshehri, Mohammad Abu Shaphe, Rashid Beg, Meshal Alshamrani.

**Software:** Ibrahim Siddig Abdelwahab, Ibrahim Elhassan, Yahya Al-Jabiri, Ahmad Sahly, Rashid Beg, Meshal Alshamrani.

**Supervision:** Ibrahim Siddig Abdelwahab, Osama Albasheer, Manal Taha, Nasir Ali, Yahya Al-Jabiri, Waleed Madkhali, Ahmad Sahly, Bassem Oraibi, Ahmed Altraif, Nasser Hakami, Mohammed Alshehri, Mohammad Abu Shaphe, Rashid Beg, Meshal Alshamrani.

**Validation:** Manal Taha, Nasir Ali, Yahya Al-Jabiri, Waleed Madkhali, Ahmad Sahly, Bassem Oraibi, Ahmed Altraif, Nasser Hakami, Mohammed Alshehri, Mohammad Abu Shaphe, Rashid Beg, Meshal Alshamrani.

**Visualization:** Manal Taha, Nasir Ali, Yahya Al-Jabiri, Waleed Madkhali, Ahmad Sahly, Bassem Oraibi, Ahmed Altraif, Nasser Hakami, Mohammed Alshehri, Mohammad Abu Shaphe, Rashid Beg, Meshal Alshamrani.

**Writing – original draft:** Ibrahim Siddig Abdelwahab, Ibrahim Elhassan, Osama Albasheer, Manal Taha, Nasir Ali, Yahya Al-Jabiri, Waleed Madkhali, Ahmad Sahly, Bassem Oraibi, Ahmed Altraif, Nasser Hakami, Mohammed Alshehri, Mohammad Abu Shaphe, Rashid Beg, Meshal Alshamrani.

**Writing – review & editing:** Ibrahim Siddig Abdelwahab, Ibrahim Elhassan, Osama Albasheer, Manal Taha, Nasir Ali, Yahya Al-Jabiri, Waleed Madkhali, Ahmad Sahly, Bassem Oraibi, Ahmed Altraif, Nasser Hakami, Mohammed Alshehri, Mohammad Abu Shaphe, Rashid Beg, Meshal Alshamrani.
